# The prevalence of using complementary and alternative medicine products among patients with pressure ulcer

**DOI:** 10.1186/s12906-022-03573-6

**Published:** 2022-03-27

**Authors:** Niloofar Karimianfard, Azita Jaberi

**Affiliations:** 1 Department of Nursing, School of Nursing and Midwifery, Shiraz University of Medical Sciences, Shiraz, Iran; 2 Community Based Psychiatric Care Research Center, Shiraz University of Medical Sciences, Shiraz, Iran

**Keywords:** Pressure ulcer, Complementary and alternative medicine, Herbal products

## Abstract

**Background:**

The use of complementary and alternative medicine (CAM) therapies has increased in recent years throughout the world and in Iran. Nonetheless, there are limited data about the prevalence of their use. This study aimed to assess the prevalence of using CAM therapies among patients with pressure ulcer (PU).

**Methods:**

This cross-sectional study was conducted in 2019–2020. Participants were 299 patients with PU conveniently selected from wound clinics and healthcare settings in Shiraz, Iran. A demographic questionnaire and the International Questionnaire to Measure Use of CAM were used for data collection. The data were analyzed using the SPSS software (v. 22.0).

**Results:**

All 299 participants completed the study. Their mean age was 59.56 ± 18.76 years. The most common CAM therapies used for PU management were herbal products (100%), vitamin supplementation therapy (45.2%), spiritual therapies (21.7%), wet cupping therapy (16.4%), leech therapy (9.4%), acupuncture (1.7%), dry cupping therapy (1.3%), and massage therapy (1.3%). The most common herbal product used for PU management was *Pistacia atlantica* gum either alone or in combination with other herbal products (15.5%). The use of CAM therapies had significant relationship with participants’ age, underlying conditions, and PU stage (*P* < 0.05) and the most significant predictor of using herbal products for PU was educational level (*P* < 0.05, OR = 5.098).

**Conclusions:**

The use of CAM therapies, particularly herbal products, for PU management is high in Iran. Quality public education and close professional supervision are needed for the safe use of these products.

## Background

Pressure ulcer (PU) is the third most costly health problem after cancer and cardiovascular disease [1]. The National Pressure Ulcer Advisory Panel (NPUAP) defines PU as localized damage to the skin usually over bony prominences due to pressure or shear and classifies it based on its severity and extent to stage 1 to 4, unstagable, and deep tissue injury [2]. The prevalence of PU is 4%–38% in intensive care units, 4.7%–31.2% in general hospital wards, 4.6%–20.7% in nursing homes, and 19% in home settings [3–6]. Two meta-analyses reported that the global prevalence of PU is 12.8% [7] and 26.6% [8]. The prevalence of PU is 14.5% in Europe, 13.6% in North America, 12.6% in the Middle East, 12.7% in South America, 9% in Australia, and 3% in Asia [7]. PU prevalence in hospitals in Iran is 5.1%–39.2% [9]. A meta-analysis reported that PU prevalence in intensive care units in Iran is 19.57% [10].

PU has many negative consequences for afflicted patients, their families, and healthcare systems. It causes pain and disability, prolongs the length of hospital stay, and increases morbidity and mortality rates [11]. PU-induced stress and altered body image also undermine individuals’ ability to participate in physical and social activities and reduce their quality of life [4]. Studies showed that sixty million people annually die due to PU-related problems [12]. PU also increases financial costs in healthcare systems [13], wastes healthcare providers’ time, and imposes heavy costs on healthcare systems and families [5]. A systematic review showed that the daily cost of PU management was 1.7–470.5 Euros per patient [14]. Estimates show that PU can increase the costs of nursing care by 50% [3].

The conventional treatments for PU include removal of pressure, position change, wound washing, infection management, use of topical ointments and dressings, establishment of appropriate nutrition, and use of advanced dressings and surgical interventions [15]. Despite the wide use of these treatments, the prevalence of PU has not significantly decreased in recent years [10], PU is still a major clinical problem and a significant burden to patients and communities, and hence, more effective therapeutic strategies are needed for PU management [15].

Complementary and alternative medicine (CAM) therapies have potentials for PU management [16]. CAM is a type of healthcare services which includes a wide range of therapies including herbal therapy, acupuncture, yoga, and massage therapy [17]. Studies showed that the prevalence of using CAM therapies for at least one time in life was 52%–69% in Australia, 33% in the United Kingdom, 66%–75% in Belgium, 49% in France, 18% in Netherlands, 20%–30% in Germany, 59% in Canada, 62% in the United States, 76% in Singapore, and 50% in Japan [18–20]. The World Health Organization estimates that 80% of the world population, i.e. around four billion people, use herbal products for at least one time in their life for the management of their health problems, though the prevalence of using different CAM therapies varies in different areas in the world depending on culture, history, educational level, and personal desires [21]. In some countries, the prevalence of using CAM therapies is as high as 80%. Healthcare authorities in different countries have heavily invested in CAM therapies due to dissatisfaction with conventional medicine therapies and their side effects, particularly in the area of managing chronic illnesses [22].

The prevalence of CAM use is also high and is progressively increasing in Iran due to factors such as their easy accessibility and low costs [23]. The official statistics of the Ministry of Health of Iran show that the rate of selling medicinal plants in Iran reached from seven billion Riyals in 1997 to 37 billion Riyals in 2001 and eighty billion Riyals in 2003 and reached from 0.3% in 2005 to 3% in recent years [24]. Meanwhile, studies showed the increasing prevalence of over-the-counter use of CAM therapies in Iran which can predispose users to significant side effects and complications. Nonetheless, there are limited data about the use, effectiveness, and costs of CAM therapies for PU management. Limited data in this area interfere with the effective and safe use of CAM therapies for PU management. Therefore, the present study was conducted to address these gaps. The aim of the study was to assess the prevalence of using CAM therapies among patients with pressure ulcer.

## Methods

This cross-sectional study was conducted in 2019–2020. Study setting was wound clinics in Shiraz, Iran, which provided wound care services to patients with different types of wounds, as well as healthcare settings in Shiraz, Iran, which provided healthcare services to patients with different health problems. Sampling was performed conveniently. Inclusion criteria were affliction by PU due to problems such as spinal plegia, stroke, accidents, or chronic conditions which required complete bed rest, and use of CAM therapies for PU management. Patients with other types of wound, such as wounds caused by burn injuries or chemicals were not included. Sample size was calculated using the Cochran formula ($n={Z}^{2} pq/{d}^{2}$) and the results of a systematic review which reported that the prevalence of PU in Iran was 19% [25]. Accordingly, with a *d* of 0.05, a *Z* of 1.96, and a *p* of 0.19, sample size was determined to be 236. Nonetheless, sample size was increased to 299 in order to compensate probable withdrawals.

Data were collected using a demographic questionnaire and the third part of the International Questionnaire to Measure Use of CAM. The items of the demographic questionnaire were on participants’ age, gender, underlying conditions, duration of affliction by PU, PU characteristics, medications, routes of taking medications, medication side effects, and reasons for using CAM therapies.

The International Questionnaire to Measure Use of CAM was developed and psychometrically evaluated by Quandt and colleagues (2009). This questionnaire has four main parts on different CAM therapies, resources which provide CAM therapies, use of herbal products and dietary supplements, and self-care activities. In each part, respondents are asked to report their use of CAM therapies within the past twelve months and at the present time, their reasons for such use, effectiveness of the used CAM therapies, and informing physicians about such use. Among the reasons for using CAM therapies are management of an acute disease, management of a chronic disease, and promotion of health or well-being. The effectiveness of the used CAM therapies is assessed on a four-point Likert scale from “I don’t know” to “Great”. Responses to each part are reported as frequency measures. This questionnaire was developed by a panel of experts and the results of a pilot study showed that its items were comprehensible for respondents, though its developers highlighted the necessity of further studies for confirming its validity and reliability in different settings [26]. Scholars in several earlier studies translated this questionnaire into German, French, Korean, Spanish, and Italian and reported its weak to acceptable face validity [27–31]. A former study in Iran translated this questionnaire into Persian and confirmed its face validity through a pilot study on twenty healthy people and its content validity through a Delphi panel of experts [32].

For data collection, we referred to the study setting together with three research assistants and completed the study instruments for eligible participants through the interview method.

### Data analysis

The SPSS software (v. 22.0) was used for data analysis. Data were described through the measures of descriptive statistics, namely mean, standard deviation, absolute frequency, and relative frequency. The relationship of using CAM therapies with demographic characteristics was assessed through the Chi-square test, while the effects of demographic characteristics on CAM use were assessed through the logistic regression analysis.

### Ethical considerations

The Ethics Committee of Shiraz University of Medical Sciences, Shiraz, Iran, approved this study (code: IR.SUMS.REC.1398.539). All methods were carried out in accordance with relevant guidelines and regulations and the Declaration of Helsinki. Necessary permissions for the study were obtained from the Research Administration of that university. Participants were ensured of data confidentiality and their informed consent for participation was secured.

## Results

In total, 299 patients with PU were assessed. Their mean age was 59.56 ± 18.76 years and they were mostly female (54.2%). The most common cause of PU was fracture (33.78%), the most common site of PU was on the sacrum or the buttocks, and the most common underlying condition was diabetes mellitus (64.2%).

Respecting the use of CAM therapies, all participants reported the use of herbal products (100%). Besides herbal products, the prevalence of using other CAM therapies was 1.7% for acupuncture, 16.4% for wet cupping therapy, 1.3% for dry cupping therapy, 1.3% for massage therapy, 9.4% for leech therapy, 45.2% for vitamin supplementation therapy, and 21.7% for spiritual therapies. None of the participants reported using homeopathy, yoga, energy therapy, and physical exercise for PU management.

The most common herbal product used for PU management was *Pistacia atlantica* gum either alone or in combination with other herbal products (15.5%). Table 1 shows the prevalence of using different herbal products for PU management. It is noteworthy to mention that all herbal products were applied topically.

**Table 1:** The frequency of using herbal products for PU management

CAM products		N	%
Honey	Alone	20	6.7
	With silver	1	0.3
	With *Oliveria decumbens*	1	0.3
	With *anbernesa*	2	0.6
	With *fennel flower*	1	0.3
	With coconut oil*, Aloe vera*	1	0.3
	With olive oil, *Prunus scoparia* gum, *chamomile*	4	1.3
	With *turmeric*	2	0.7
	With *turmeric, Aloe vera, Rosa damascena* oil*, Amygdalus communis L. var. dulcis*	1	0.3
	With sesame oil, *Mummy*	1	0.3
	Total	34	11.1
*Pistacia atlantica* gum	Alone	11	3.7
	With Ghee	33	10.9
	With *Pinus eldarica* gum	1	0.3
	With *Pistacia atlantica* leaves	1	0.3
	With *Pistacia atlantica* wood	1	0.3
	Total	47	15.5
*Teucrium polium* and *anbernesa*	Alone	7	2.3
	With *chamomile*	7	0.3
	With *Artemisia vulgaris*	1	0.3
	Total	9	2.9
*Aloe vera*		14	4.7
*Glycyrrhiza glabra*		15	5
Henna with *Ziziphus spina-christi*, milk, lemon juice, *Pistacia atlantica* gum, and Ghee		5	1.5
*Mummy*	Alone	7	2.3
	With *Pistacia atlantica* gum and Ghee	1	0.3
	With *Trigonella foenum-graecum* and *Cuminum cyminum*	1	0.3
	With *Peganum harmala*	1	0.3
	Total	10	3.2
Egg	Egg albumin	1	0.3
	Egg yolk	1	0.3
	Egg yolk with burned *Lens culinaris*	1	0.3
	Egg yolk with olive oil	1	0.3
	Egg yolk with *Prunus scoparia* gum	1	0.3
	Total	5	1.5
Burned or powdered *Lens culinaris*		8	2.7
Other		146	48.8

The main reasons for using CAM therapies were their greater effectiveness (49.8%), their greater effectiveness and lower costs (15.7%), and their greater effectiveness and easier accessibility (9%). Most participants had acquired their information about CAM therapies through their relatives (61.87%) and online search using mobile phone (31.1%). We had also asked participants whether they experienced the symptoms of wound infection, including exudates, discoloration, burning, pain, or fever, at one day, one week, two weeks, three weeks, and one month after the use of herbal products. As Table 2 shows, these symptoms were most common at one day after use (4.6%) and one week after the use (2.2%), while they were rare at two weeks, three weeks, and one month after the use (less than 1%).

**Table 2:** The symptoms of PU infection after using herbal products

Symptoms	Exudates		Discoloration		Burning		Pain		Fever	
Time										
	N	%	N	%	N	%	N	%	N	%
One day after	3	1	3	1	7	2.3	1	0.3	0	0
One week after	1	0.3	1	0.3	4	1.3	1	0.3	0	0
Two weeks after	1	0.3	0	0	2	0.7	0	0	0	0
Three weeks after	1	0.3	0	0	1	0.3	0	0	0	0
One month after	1	0.3	0	0	1	0.3	0	0	0	0

As Table 3 shows, participants used CAM therapies mainly for a chronic disease (71.9%) and considered them effective (54.5%). Moreover, only 21.1% of them reported that their physicians were aware of their use of CAM therapies.

**Table 3:** Reasons for using CAM therapies, their effectiveness, and physician’s awareness of their use

Variables		N	%
Reasons for use	Managing acute disease (less than one month)	23	7.7
	Managing chronic disease (more than one month)	215	71.9
Effectiveness	Great	163	54.5
	Relative	76	25.4
Physician’s awareness	Yes	63	21.1
	No	178	59.5

The use of CAM therapies had no significant relationship with participants’ gender, educational level, occupation, and PU duration (*P* > 0.05). However, the use of CAM therapies had significant relationship with their age, underlying conditions, and PU stage so that the use of CAM therapies was greater among participants with diabetes mellitus, older age, and stage II PU (*P* < 0.05; Table 4).

**Table 4:** The relationship of using CAM therapies with demographic characteristics

Characteristics		Mean ± SD or N (%)		*P* value
Age (years)		59.56 ± 18.76		< 0.001
Gender	Female	162	54.2	0.151
	Male	131	43.8	
Underlying condition	Diabetes mellitus	192	64.2	< 0.001
	Cardiovascular disease	97	32.5	
	Renal disease	3	1	
	Liver disease	1	0.3	
	Other	6	2	
Educational level	Illiterate	103	34.4	0.142
	Secondary	140	47.2	
	Bachelor’s	18	6	
	Master’s	1	0.3	
Occupation	Housewife	154	51.5	0.85
	Employee	1	0.3	
	Retired	94	31.4	
	Unemployed	12	4	
PU stage	1	5	1.7	0.001
	2	208	69.6	
	3	58	19.4	
	4	7	2.3	
PU duration	One week	63	21.1	0.054
	Two weeks	103	34.4	
	3 weeks	40	13.4	
	4 weeks	27	9	
	5 weeks	47	16.1	

The results of the logistic regression analysis revealed that the most significant predictor of using herbal products for PU was educational level (*P* < 0.05, OR = 5.098; Table 5).

**Table 5:** The results of the logistic regression analysis to determine the predictors of CAM therapy use

Independent variable		Sig	OR	95% CI for OR	
				Lower	Upper
Step 1	Age	0.272	1.026	0.980	1.074
	Gender	0.578	2.946	0.066	132.450
	Educational level	0.028	5.098	1.190	21.847
	Occupation	0.397	0.662	0.254	1.722
	PU stage	0.214	0.514	0.180	1.468

## Discussion

Study findings showed that in addition to conventional therapies, participants used CAM therapies, particularly herbal products. A former study also reported that the most prevalent CAM therapies used in chronic conditions were herbal products and spiritual therapy [32]. Some herbal products used in the present study, such as *anbernesa*, were burned and PU was smoked with them. Female donkey excrement, also known as AnbarNesa (or AnbarNasara), is gathered after labor and in the early spring. AnbarNesa smoke was also commonly utilized in Iranian traditional medicine to treat wounds, chickenpox blisters, oral inflammatory diseases such as aphthous ulcers, and inflammations such as otitis media and externa [33]. Such products have traditionally been used in the Iranian Traditional Medicine for managing infections, such as genitourinary tract infections, and recent studies confirmed their effectiveness in wound infection management and hence, they are currently used for wound dressing [33]. Our participants had also used egg albumin for PU management. Some previous studies also reported the use of egg albumin in hydrogel wound dressing [34, 35].

We also found that participants had used honey for PU management, either alone or in combination with other CAM products. Numerous studies have been conducted into the effects of honey on wound healing [36, 37]. Honey has antimicrobial effects, regulates the immune system, reduces edema and exudates, and hence is used for managing leishmaniosis wounds, surgical wounds, traumatic injuries, and chronic wounds such as PU [38].

Our participants also reported using *Pistacia atlantica* gum for PU management. This plant, its fruit, and its gum are used to treat a variety of disorders in Iran, either orally or topically. Previous research in Persian medicine has revealed that this herb can help with wound healing and the pain associated with other skin diseases [39]. Due to enhanced angiogenesis and increased bFGF and PDGF, Tanideh and colleagues discovered that the resin found in gum tissue is beneficial in treating burn wounds [40]. *Pistacia atlantica* is effective in managing deep wounds and preventing refractory wounds [41]. Study participants had also used *Teucrium polium* and *Aloe vera* for PU management. *Aloe vera* has known positive effects on wound healing [42]. Moreover, *Teucrium polium* and *Aloe vera* were reported to have positive effects on dermatologic disorders such as dandruff [43].

A very small number of participants had used *Broadleaf plantain* for PU management. Studies showed that due to its anti-inflammatory effects, Broadleaf plantain has beneficial effects on deep, infectious, chronic, and progressive wounds and burn injuries [44]. Some participants had used CAM therapies through burning, warming, or grinding them. For example, some of them had warmed egg yolk up to the extraction of its oil and then, applied it to PU. We did not find any study to support such use of CAM therapies. Some studies in Iran, particularly in southern areas of Iran, reported the use of herbal products such as *Artemisia vulgaris*, *Teucrium polium*, and *Plantago ovate* for wound management, though interventional studies are still needed to assess their mechanisms of action [45]. Another study reported the traditional use of *coconut oil*, *Gentiana lutea, Commiphora Mukul, Teucrium polium, Punica granatum, Aloe vera, Adiantum capillus-veneris, Dutchman’s pipe,* and *Potentilla recta* for wound management. Moreover, that study reported that while some herbal products has antimicrobial, anti-inflammatory, and antioxidant effects, there are no empirical data about their effects on wound healing. These products are *Pistacia atlantica* gum*, camphor, chamomile, and rhamnus* [16]. Our participants’ lack of knowledge about some herbal products highlights the importance of providing them with quality education by CAM specialists.

We also found that around half of the participants (45.2%) used vitamin supplements. A meta-analysis showed that using zinc-containing formula and oral supplements for eight weeks significantly improved PU healing [46]. On the other hand, none of our participants had used homeopathy, yoga, physical exercise, and energy therapy for PU management probably due to their limited knowledge about these CAM therapies. Moreover, one fifth of the participants reported the use of spiritual therapies such as praying. This rate is greater than the rates reported in previous studies in other countries [47] probably due to the religious beliefs of people in Iran [48]. Our findings also showed that 16.4% of participants had used wet cupping therapy. This rate is also greater than the rates reported in previous studies [30, 49]. The higher rate of using wet cupping therapy in the present study compared with former studies is attributable to the differences among studies respecting their participants’ characteristics, health status, and underlying conditions. Cupping treatment is an ancient form of alternative medicine in which a therapist suctions the skin with specific cups for a few minutes. Cupping is used to improve local blood and lymph circulation as well as relieve uncomfortable muscular tension. A glass cup is used in each of the various cupping techniques to induce suction over a painful spot. Cups are put to the undamaged skin in dry cupping, whereas in wet or bloody cupping, the skin is incised before the cups are administered [50]. Wet cupping therapy reduces the level of fibrinogen and alleviates inflammation and thereby, has potential positive effects on PU healing [51].

Our participants reported that their main reasons for using CAM therapies were their easy accessibility, lower costs, and greater effectiveness. Moreover, the main reason for using herbal products was to manage chronic conditions. These findings are in line with the findings of previous studies in Iran [9, 24]. However, most participants reported the use of CAM therapies without informing their physicians. This practice may expose them to problems such as drug interactions and medication side effects and highlights the importance of quality public education about using CAM therapies under professional supervision. Informing physicians about the use of CAM therapies facilitates recovery and helps physicians and patients better manage the side effects of therapies.

We also found that the main sources of our participants for obtaining information about CAM therapies were relatives and online search using mobile phone. This finding denotes that they had no information about the possibility of wound infection after using products such as unprocessed honey and *anbernesa*. Unprocessed honey and *anbernesa* may contain microorganisms and cause infection [33]. Quality public education about CAM therapies, particularly about the products of Iranian Traditional Medicine, should be provided through short message service, mobile phone applications, and multimedia programs in order to improve public awareness about these therapies and products.

An important finding of the present study was the low prevalence of PU infection despite the affliction of some participants by serious underlying conditions such as diabetes mellitus. Meanwhile, our findings showed that most participants had not used herbal products with known positive effects on wound healing such as *Punica granatum, coriander, Rosa damascena, cinnamon, gum Arabic, grapevines, cinnamon extract*, and the mucilage of *Broadleaf plantain* and *Plantago ovate* [52].

Concerning the findings, follow-up studies with doctors and nurses who specialize in wound care would be interesting to assess their knowledge of what their patients are using for PU management and to address the disconnection between patient confidence in prescribed medication and self-medication. Clinical investigations of herbal and other CAM PU treatment strategies are one of the study's implications. Furthermore, doctor and nurse education on the topic, as well as the formulation of national policy on the use of evidence-based CAM modalities for safe and cost-effective PU care, are both necessary.

## Conclusions

This study shows the high prevalence of using CAM therapies, particularly herbal products, for PU management. Such use is mainly over-the-counter and without informing treating physicians and CAM specialists. Therefore, quality public education through mass media is recommended to improve public knowledge about CAM therapies, their uses, and their side effects. Improving healthcare providers’ and lay people’s knowledge about CAM therapies and the appropriate use of cost-effective CAM therapies in PU management can reduce the need for imported dressings which are expensive and poorly accessible.

## Data Availability

The datasets used and/or analyzed during the current study are available from the corresponding author on reasonable request.
