# Correction to: The prevalence of using complementary and alternative medicine products among patients with pressure ulcer

**DOI:** 10.1186/s12906-022-03588-z

**Published:** 2022-04-21

**Authors:** Niloofar Karimianfard, Azita Jaberi

**Affiliations:** 1 Department of Nursing, School of Nursing and Midwifery, Shiraz University of Medical Sciences, Shiraz, Iran; 2 Community Based Psychiatric Care Research Center, Shiraz University of Medical Sciences, Shiraz, Iran

**Correction to: BMC Complement Med Ther 22, 91 (2022)**

**https://doi.org/10.1186/s12906-022-03573-6**

Following publication of the original article [1], the authors identified an error in the correspondence affiliation of Dr. Azita Jaberi in the first page of the article.

The incorrect affiliation is: Department of Nursing, School of Nursing and Midwifery, Shiraz

University of Medical Sciences, Shiraz, Iran

The correct affiliation is: Community Based Psychiatric Care Research Center, Shiraz University of Medical Sciences, Shiraz, Iran

The original article [1] has been updated.
